# Multispectral Face Recognition Using Transfer Learning with Adaptation of Domain Specific Units

**DOI:** 10.3390/s21134520

**Published:** 2021-07-01

**Authors:** Luis Lopes Chambino, José Silvestre Silva, Alexandre Bernardino

**Affiliations:** 1 Instituto Superior Técnico, Universidade de Lisboa, 1049-001 Lisbon, Portugal; luis.chambino@tecnico.ulisboa.pt (L.L.C.); alex@isr.tecnico.ulisboa.pt (A.B.); 2 Portuguese Military Academy, Rua Gomes Freire, 1169-203 Lisbon, Portugal; 3 Military Academy Research Center (CINAMIL), Rua Gomes Freire, 1169-203 Lisbon, Portugal; 4 Laboratory for Instrumentation, Biomedical Engineering and Radiation Physics (LIBPhys-UC), Rua Larga, 3004-516 Coimbra, Portugal; 5 Institute for Systems and Robotics (ISR), 1049-001 Lisbon, Portugal

**Keywords:** facial recognition, multispectral images, infrared, presentation attack detector

## Abstract

Facial recognition is a method of identifying or authenticating the identity of people through their faces. Nowadays, facial recognition systems that use multispectral images achieve better results than those that use only visible spectral band images. In this work, a novel architecture for facial recognition that uses multiple deep convolutional neural networks and multispectral images is proposed. A domain-specific transfer-learning methodology applied to a deep neural network pre-trained in RGB images is shown to generalize well to the multispectral domain. We also propose a skin detector module for forgery detection. Several experiments were planned to assess the performance of our methods. First, we evaluate the performance of the forgery detection module using face masks and coverings of different materials. A second study was carried out with the objective of tuning the parameters of our domain-specific transfer-learning methodology, in particular which layers of the pre-trained network should be retrained to obtain good adaptation to multispectral images. A third study was conducted to evaluate the performance of support vector machines (SVM) and k-nearest neighbor classifiers using the embeddings obtained from the trained neural network. Finally, we compare the proposed method with other state-of-the-art approaches. The experimental results show performance improvements in the Tufts and CASIA NIR-VIS 2.0 multispectral databases, with a rank-1 score of 99.7% and 99.8%, respectively.

## 1. Introduction

Nowadays, many biometric facial recognition systems use images of the visible spectral band. When compared to other types of biometric traits, such as iris, fingerprint, vein signature and voice recognition, facial recognition presents some advantages, such as more easily detecting a person’s characteristics, and being non-invasive [1,2].

Systems that use only the visible spectral band have multiple caveats, such as intolerance to occlusion, sensitivity to face pose variations, need for human cooperation, and lack of robustness to luminosity changes. As a result, it is necessary to complement current facial recognition systems with the use of other biometric sensors (e.g., iris or voice) or exploit the information contained in other spectral bands to mitigate these problems [3].

A system that uses multiple spectral bands is called multispectral. Compared to facial recognition systems that use the spectral bands of the visible spectrum, the multispectral systems typically have a higher accuracy in facial recognition, thus allowing higher safety levels in the access to critical facilities where the access is only permitted to authorized people, e.g., hospitals, schools, laboratories, and military buildings [3]. By developing better facial recognition systems it is possible to ensure more reliable and robust access control, thus protecting property and increasing the safety of people.

The infrared electromagnetic spectrum can be divided into four distinct spectral bands, namely the Near Infrared (NIR), Short Wavelength Infrared (SWIR), Mid Wavelength Infrared (MWIR) and Long Wavelength Infrared (LWIR). These spectral bands have been applied successfully in facial recognition systems as a complement to the visible spectrum [1,3]. The use of infrared electromagnetic spectrum in facial recognition systems has several advantages. Infrared is not detectable by the human eye and, at the same time, it is not very sensitive to luminosity changes. When used in combination with infrared LED illumination, infrared cameras can be used in video surveillance during the night to detect people without being noticed [4,5].

As the NIR and SWIR spectral bands are close to the visible spectral band in the electromagnetic spectrum, it is possible to take a machine learning method already trained with images of the visible spectrum and adapt it to the NIR and SWIR bands [6]. The MWIR and LWIR spectral bands, commonly known as “thermal”, allow the use of facial recognition systems at night, when the luminosity is reduced or even inexistent.

Existing multispectral face datasets have numbers of samples much lower than the datasets of face images in the visible spectrum. Thus, training neural network models in multispectral datasets alone is limited by the low amount of available data. Instead, we propose a transfer-learning approach to multispectral face recognition that exploits the existence of face recognition networks pre-trained in large databases of images in the visible spectrum. These pre-trained networks are fine-tuned in the multispectral datasets by adapting the initial network layers that are more specific to different spectral characteristics of the images in the visible and multispectral domains. The proposed method compares favorably to the state-of-the-art in the typical benchmark datasets.

This work is organized as follows. In Section 2 we present the state-of-the-art on multispectral facial recognition methods, including the most used evaluations metrics and public multispectral databases. In Section 3 the proposed methodologies for multispectral facial detection are described. In Section 4 we present the experimental setting and results obtained, including their respective analysis and discussion. Finally, Section 5 concludes the paper. 

## 2. State-of-the-Art

This section outlines a systematic review of articles in multispectral facial recognition and analyses their distribution by year and field of study. The study was conducted in June 2020 with the help of the Web of Science database. All articles published in scientific journals with impact factors from January 2000 to June 2020 were selected (works published in conferences were not considered) [7]. 

Through a specific set of search parameters, 283 articles in 132 scientific journals were found. Narrowing down the search to articles that use at least two spectral bands (e.g., VIS-NIR, VIS-LWIR, VIS-NIR-LWIR, NIR-LWIR) the total number of articles was reduced to 47. These papers were considered the most relevant to our work.

An analysis of these articles was carried out taking into consideration the multispectral databases and evaluation metrics used. It was concluded that the most commonly used database was the CASIA NIR-VIS 2.0 [8], used 15 times in the 47 surveyed papers [7].

The most used evaluation metric was the rank-1, i.e., the percentage of predicted identities high highest score (rank-1) that correspond to the correct identity.

Through the systematic analysis, the most relevant papers were grouped into five approaches: feature representation methods, coupled subspace learning, image synthesis, fusion, and deep neural networks. The most used method, in 32% of the articles analyzed, was deep neural networks.

The feature representation methods seek to extract the characteristics that are more invariant to the spectral band used. Through the extraction of facial features (e.g., contours, corners, eyes, and mouth) it is possible to encode in an efficient way the information contained in the image.

Methods that project the features of different spectral bands into a common subspace are known as coupled subspace learning methods. This subspace allows the identification of the information that is common to the different spectral bands used.

Image synthesis methods transform an image from one spectral band to another spectral band. These methods allow synthesizing an image in the visible spectral band using an image from another spectral band (e.g., LWIR), and then apply a facial recognition method to the synthesized image.

The overall performance of multispectral facial recognition systems can be improved by combining (fusing) several types of images. The most relevant image fusion methods applied in facial recognition are feature fusion and score fusion, that can be used individually or in combination. The feature fusion method combines the features obtained from several images into a feature vector. The score fusion method improves the overall rating performance by combining the classifications obtained from multiple images.

Currently, the neural networks most used in facial recognition are the deep convolutional neural networks (DCNNs). These networks have a higher number of layers than the traditional neural networks. DCNNs are composed of several layers of convolution, activation, and pooling. The repetition of these layers allows the identification of efficient feature representations with high predictive power, denominated embeddings.

## 3. Materials and Methods

**Figure 1:**
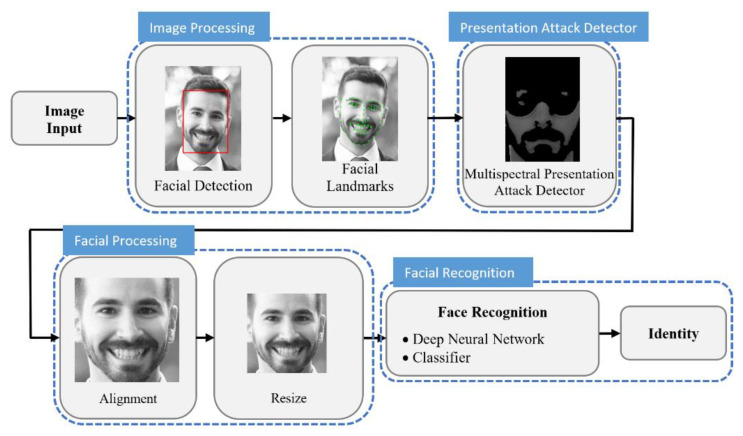
Block diagram of the proposed multispectral facial recognition system.

This section presents the proposed multispectral facial recognition system. Figure 1 shows the block diagram of the proposed methodology.

It follows a quite classical pipeline except for two modules that yield the main contribution of this work: the presentation attack detector and the deep neural network classifier. Details of these two modules are presented later.

The proposed method begins with the acquisition of multispectral images (e.g., visible and infrared). These images can be obtained by several mono-spectral cameras, each acquiring an image of a particular spectral band, or by imaging equipment capable of obtaining images at various spectral intervals. The only requirement at this stage is that the images are obtained at the same instant of time, so that they correspond to the same person and the same conditions of luminosity and pose.

The next steps are to convert the images to greyscale, to detect the human faces, and to extract the facial landmarks (e.g., eyes, nose, and mouth). The module that performs this task is represented in the block diagram of Figure 1 as the image processing module.

The proposed facial recognition system includes a module for detecting and warning potential presentation attacks, called the presentation attack detector module. This module takes advantage of all available multispectral images to perform skin detection, thus preventing the facial recognition system from possible presentation attacks.

In the next module, denoted facial processing, facial landmarks obtained in the image processing module are used to align the face. The image is rotated so that the facial landmarks of the eyes are horizontally aligned. Then, the image is resized to 144 × 144 pixels. The main objective of this module is to normalize the image before introducing it to the DCNN. 

Finally, the facial recognition module receives the normalized images and performs the identification of the person.

### Facial Recognition

The facial recognition module is composed of two main components: a DCNN and a classifier. The purpose of the DCNN is to extract embeddings representative of the person to be identified. Then, the classifier will use these embeddings to determine the identity of the person.

**Figure 2:**
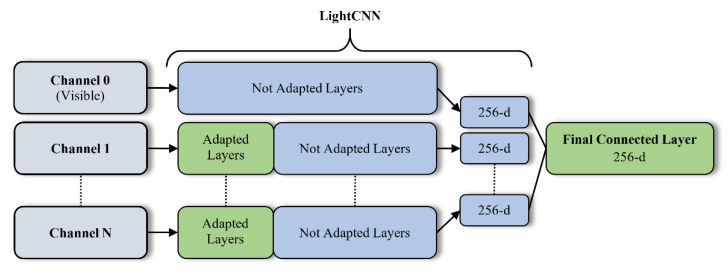
DCNN proposed architecture.

The DCNN architecture used to extract the embeddings of a facial image is shown in Figure 2, following the work of George [9] and Pereira [10]. This architecture allows the use of several (N) channels, allocating to each channel a spectral band, or spectral range (if several spectral ranges are being used in the same band). Furthermore, each channel can leverage a backbone DCNN pre-trained in a large dataset of facial images in the visible spectrum. This allows to apply transfer learning techniques, i.e., use a small dataset of labeled images of each channel to adapt the corresponding backbone DCNN to the spectral characteristics of that channel. 

The backbone DCNN used in this work is the LightCNN [11]. This DCNN stands out from other similar DCNNs because it employs Max-Feature Map (MFM), an extension of the Maxout activation function, in its base architecture, as an alternative to the rectified linear unit (ReLU) activation function. Through this activation function, LightCNN obtains a reduced number of parameters. This network takes as input greyscale images of size 128 × 128 pixels, and outputs embeddings of 256 dimensions, representative of the identity of the person. 

Different layers in the LightCNN [11] are adapted to adjust the model to the spectral bands of the corresponding channel. If the channel is assigned to the visible spectral band, the DCNN is not modified, since the domain of use is identical to the domain where the DCNN was pre-trained. For the other channels, the transfer learning methodology is applied. Reusing the weights of a pre-trained DCNN in a face recognition database with a high number of facial images, is a way to prevent overfitting the training in a new domain, given the limited number of training multispectral images in most existing datasets [10].

To fuse the classifications of all channels, a Fully Connected Layer (FCL) is added at the end of the network. This layer is fed with the vector of dimension N × 256, resulting from the concatenation of the individual channel embeddings. The output of the FCL is a final 256-d embedding, that will feed the classifier block to recognize the input face.

Figure 2 shows a generic case of using the proposed network with N channels. The layers that are adapted are colored green, while the frozen (not adapted) layers are in blue. Channel 0 is assigned to the visible spectral band, so it is not adapted.

After obtaining the final 256-d embedding, it is necessary to classify it and obtain the corresponding identity of the person in the image. Several classifiers were tested to find out which is the most suitable to classify the 256-d embeddings extracted by the proposed network. The most frequently used classifiers were tested: the SVM with linear or radial basis function (RBF) kernel, and the kNN [12].

**Figure 3:**
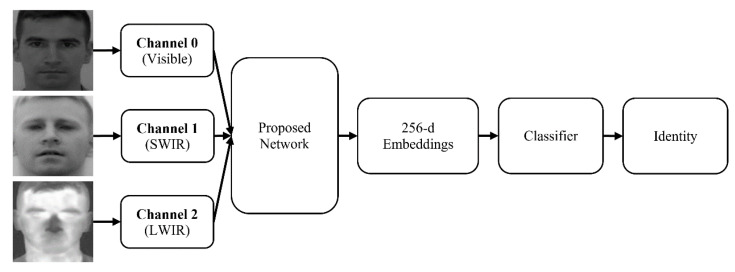
Architecture of the implemented facial recognition module.

Figure 3 shows a summary scheme that exemplifies the facial recognition module, from the input of multispectral images, for each channel, to the identification of the identity of the person present in these images.

## 4. Results and Discussion

This section describes the multispectral databases used and the tests carried out to assess the performance of the proposed methods. In a first test, we present and evaluate the presentation attack detector. Then, we study which layers of the LightCNN should be adapted to improve the quality of the face embeddings. Then, we study which classifiers are better to classify a person’s identity through the 256-d embeddings. Finally, the best classifier is compared with the state-of-the-art methods in multispectral face recognition.

All the experimentations were carried out on the Ubuntu operating system, version 18.04, with a graphical processing unit (GPU) NVIDIA^®^ GeForce^®^ GTX 1650 with 4GB of dedicated GDDR6 random access memory (RAM). All code was developed in the programming language Python 3.7.

### 4.1. Multispectral Databases

To evaluate the proposed algorithms, three multispectral databases were used: Tufts [13], CASIA NIR-VIS 2.0 [8] and our own AM database, a multispectral dataset acquired at the Portuguese Military Academy (AM). Before the multispectral databases were used, a cleaning and a pre-processing step was performed. The cleaning step aimed at excluding from the databases the images that are unusable (e.g., corrupt or blurred). The pre-processing step consisted in the offline execution of the image processing and facial processing blocks of Figure 1, to precompute all steps that are common across the experiments: the face detection, facial alignment, cropping and resizing the images to fit the DCNN input size. In some images the automatic facial detection was unable to locate the faces, so it was necessary to make a manual facial detection. 

From the Tufts database [13] were collected all images with VIS, NIR and LWIR spectral bands. The cleaning step excluded 53 facial images of 4 people, resulting in a total of 7675 facial images of 109 people.

CASIA NIR-VIS 2.0 [8] is composed of two spectral bands, VIS and NIR, with 17,489 facial images of 715 people.

**Figure 4:**
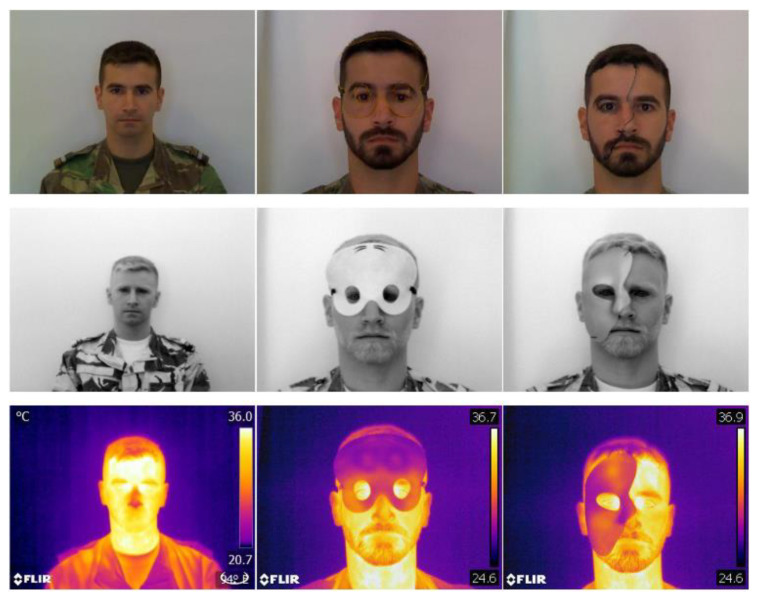
Example of images from our AM multispectral database.

Our AM database is composed of three spectral bands, VIS, SWIR and LWIR. This database was built to evaluate our presentation attack detector module. During the construction of this database several masks of different sizes and materials were used to simulate different presentation attacks. Figure 4 represents some of the images included in this multispectral database, made at the Portuguese Military Academy.

### 4.2. Presentation Attack Detector

The tests performed on this module aim to study the advantages of using multispectral images in presentation attack detection.

A skin detector was developed to support the attack presentation detection module. It takes advantage of all available spectral bands to perform the skin detection. It receives the facial landmarks extracted in the image processing module (see Figure 1). If the number of facial landmarks in the skin region is less than 75% of the total, then a presentation attack is detected. To illustrate the advantages of the proposed multispectral skin detection, a comparison was made with a similar approach applied using visible spectrum images in the YCbCr and the HSV color spaces.

#### 4.2.1. Skin Detector

The skin detector performs a pixel-level detection. In the first step, the normalized difference between all facial channels is computed, *d*[*g_a_*, *g_b_*]:

(1) $$d\left[g_{a},g_{b}\right]=\left(\frac{g_{a}-g_{b}}{g_{a}+g_{b}}\right)$$

where *g* corresponds to the pixel intensity value for channels *a* and *b*, with 1 ≤ *a* ≤ *b* ≤ *n*, where *n* corresponds to the number of channels available in the presentation attack detector module. The normalized differences are in the range −1 ≤ *d*[*g_a_*, *g_b_*] ≤ + 1. The skin detector uses the normalized difference values to classify the pixels as “skin” or “not skin”. The range of values chosen to classify pixels as “skin” or “not skin” was defined empirically for images in the VIS, SWIR and LWIR spectral bands: (76, 51, 65) < (*d*[*g*_1_, *g*_2_], *d*[*g*_1_, *g*_3_], *d*[*g*_2_, *g*_3_]) < (131, 140, 127). A binary map is produced to represent the skin pixels, where “1” equals “skin”, and “0” equals “not skin”. This binary map is used to support the computation of the number of facial landmarks that are considered “skin” for the presentation attack detector. 

**Figure 5:**
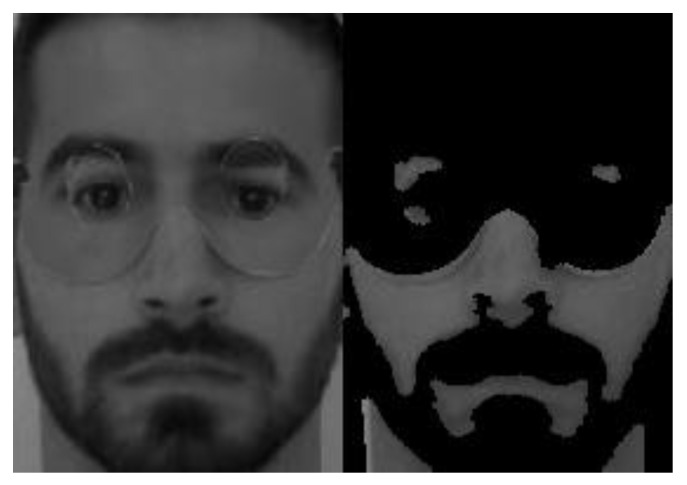
Original image (**left**) and the binary map (**right**), after applying the proposed skin detector.

In Figure 5 we show an example of the skin detector output. The image on the left corresponds to the original image. The image on the right corresponds to the binary map after applying the proposed skin detector; the black region corresponds to what was classified as “not skin”.

**Figure 6:**
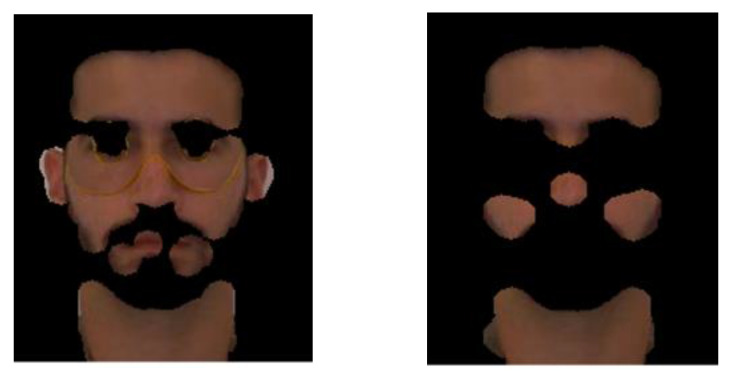
Results obtained with YCbCr (**left**) and HSV (**right**) classifiers.

To validate our proposal, we applied a similar approach to images of the visible spectrum in the YCbCr and HSV color spaces, using skin color thresholds reported in the literature (see Figure 6).

A first analysis of Figure 5 and Figure 6 shows that the multispectral skin detector can make a better skin detection, when compared to YCbCr and HSV skin detectors. The multispectral detector was the only one that could distinguish the real skin from the fake mask skin.

#### 4.2.2. Attack Detector

At this stage, the facial landmarks extracted from the image processing module are used together with the skin binary map to detect the presence of a presentation attack. For this detection, the percentage of facial landmarks that are considered “skin” is computed. If the percentage of facial landmarks is less than 75%, then our multispectral facial recognition system is facing a presentation attack. We applied this rule to the AM database images, using the multispectral, YCbCr and HSV skin detectors. With only the visible spectral bands a presentation attack detection rate (i.e., correct decision on a presentation attack) of 13% was achieved, given that the used classifiers were not able to make a correct discrimination of the human skin from the mask.

When the multispectral presentation attack detector that uses VIS, SWIR and LWIR spectral bands was applied, better results were achieved: the presentation attack detection rate was 83%. The multispectral skin detector can make a correct discrimination of the mask, as shown in Figure 5.

### 4.3. Facial Recognition 

The facial recognition module starts from the results of the pre-processing step: a normalized face image, cropped and scaled to the dimension of the DCNN. This image is processed by the DCNN to extract its 256-d embedding vector. Then, the embedding vector can be used with a classifier to obtain the identity of the person present in the images.

Several tests were carried out to find out which layers of the DCNN should be fine-tuned with domain specific data and which type and hyperparameters of the classifiers provide the best performance.

The images of each database (Tufts and CASIA NIR-VIS 2.0) were divided into three sets: training, validation, and testing. The percentage of images for the training set was 64%, for the validation set 16%, and finally 20% for the test set. Was performed a stratified division in the database so that each person has an equitable number of facial images in each set.

#### 4.3.1. Training Procedure

Data augmentation was used to obtain a more generalized model. In the training set horizontal random mirroring and random cropping augmentations were used to resize the image to a resolution of 128 × 128 pixels (to allow random cropping data augmentation the training images were resized to 144 × 144 in the facial processing module, instead of 128 × 128). For the validation set, a crop of 128 × 128 in the center was performed to meet the LightCNN [11] input size.

The Cross Entropy loss (CE) was used for the DCNN training. As the DCNN was implemented in Pytorch, the Cross Entropy loss function combines SoftMax logarithmic (LogSoftMax) and negative log likelihood (NLLLoss) in a single loss function.

The batch size was selected so that the number of images per batch was as large as possible to fit in the graphic processing unit (GPU) memory during the training phase. However, it was necessary to ensure that the number chosen for the batch of images was an exponent of 2 (note that, this limitation is due to the alignment of the virtual processors in the physical processors of the GPU), as suggested by Mishkin [14] and Goodfellow [15]. 

Table 1 summarizes the used parameters, for each multispectral database, for training the DCNNs of the proposed architecture.

#### 4.3.2. Adapted Layers

Several tests were performed to estimate which layers are suitable to adapt in the LightCNN architecture [11]. In all experiments, the weights were initialized from the original LightCNN model (the model used is available, for Pytorch, in the following Github repository: https://github.com/AlfredXiangWu/LightCNN (accessed on 30 June 2021)) [11]. The nomenclature of the network layers follows the original LightCNN paper [11]. LightCNN has 29 layers. Among all layers, 9 sets of layers stand out: the first convolutional layer together with the first MFM, denominated *Conv1*, 4 sets denominated of *Group*, which constitute the layers between the pooling layers, and the remaining 4 layers denominated *Block*, consisting of a block of convolutional layers at the beginning of each *Group*. The notations used in the combination of the adapted layers are the following:

- **FCL** (This layer is not part of the original LightCNN and was added in our architecture to fuse the outputs of the DCNNs of the multiple channels, so that the output of the architecture results in a 256-d embedding, see Figure 2): Only final connected layer is adapted;
- **Conv1-FCL** (**{1–1} + FCL**)**:** The first convolutional layer is adapted in conjunction with MFM and FCL;
- **Conv1-Block1- FCL** (**{1–2} + FCL**)**:** Block of residual neural networks is adapted together with the previous layers;
- **Conv1-Block1-Group1- FCL** (**{1–3} + FCL**)**:** Adapts Group-1 together with the previous layers;
- **Conv1-N- FCL** (**{1-N} + FCL**)**:** Adapts layers 1 to N together with the FCL;
- **All Layers:** All layers of LightCNN and FCL are adapted.

The number of epochs used during training was 10 and 50 for the Tufts [13] and CASIA NIR-VIS 2.0 [8] databases, respectively. After the training, the 256-d embeddings were extracted from each image of the multispectral databases. To evaluate each model an SVM-Linear classifier was used to classify the 256-d embeddings.

For both databases, the best results were achieved when the initial layers ({1–3} + FCL) were adapted. As more layers were adapted, the performance started to deteriorate. The obtained results in this setting were rank-1 metrics of 99.7% and 99.8% for the Tufts [13] and CASIA NIR-VIS 2.0 [8] multispectral databases, respectively.

#### 4.3.3. Hyperparameter Analysis

With the best model obtained in the previous section, the 256-d embeddings of all faces were extracted. To classify these embeddings, we trained and evaluated the performance of SVM classifiers (with a linear and RBF kernel) and kNN classifiers.

To choose the hyperparameters of each classifier, stratified cross-validation (SCV) was used, to deal with the unbalance of the database by forcing the number of images per person to be identical, as described by Forman [16] and Tsamardinos [17]. For the SCV the training and validation sets were unified in a single set from which multiple training and validation partitions were generated. During the training phase of the classifier after the best hyperparameters were determined, only the original training set was used (i.e., without the original validation set).

In SCV the number of images per person is limited by the person who has the smallest number of images. This number is 5 for the Tufts database [13] and 4 for the CASIA NIR-VIS 2.0 database [8].

The hyperparameter tuned for the SVM-Linear classifier was the regularization parameter (C) that indicates the degree of importance given to incorrect classifications. The range of values studied for this hyperparameter was 10^−10^ ≤ *C* ≤ 10^+5^, in a base-10 logarithmic scale.

For the SVM-RBF classifier the following hyperparameters were tuned: the smoothing parameter (C) and the kernel coefficient (Υ). The kernel coefficient hyperparameter aims at defining how neighbor data points influence each other according to their proximity. The range of values studied for the C hyperparameter was 10^−4^ ≤ *C* ≤ 10^+7^, and for Υ was 10^−10^ ≤ Υ ≤ 10^+2^, both in a base-10 logarithmic scale.

Finally, for the kNN classifier, the hyperparameter to be tuned was the number of close neighbors (k). This hyperparameter influences the number neighbors that are considered to classify a point. The range of values analyzed for the k hyperparameter was 1 ≤ *k* ≤ 25.

From the results of this analysis, shown in Table 2 and Table 3, it can be observed that SVM classifiers, regardless of the kernel used, obtain a higher rank-1 score, namely of 99.89% and 99.86% for Tufts [13] and CASIA NIR-VIS 2.0 [8] databases, respectively. These tables also show that the best value for the number of neighbors for the kNN hyperparameter is 1, independently of the multispectral database used.

#### 4.3.4. Comparison with State-of-the-Art Methods

To evaluate the generalization ability of the trained networks and classifiers, it is necessary to evaluate their performance in the test sets of each multispectral database. We report the results using the cumulative correspondence characteristic curve (CMC). This curve traces the identification rate on the ordinate axis and the rank-N on the abscissa axis.

Using the values in rank-1 and the values obtained for different classifications (e.g., CMC curve) it is possible to conclude on the best classifier for each multispectral database.

##### Results Using the Tufts Database

After evaluating the test set, from the Tufts database, with the classifiers (i) SVM-Linear, (ii) SVM-RBF and (iii) kNN, the following values were obtained in rank-1: (i) 99.7%, (ii) 99.2% and (iii) 99.2%. 

**Figure 7:**
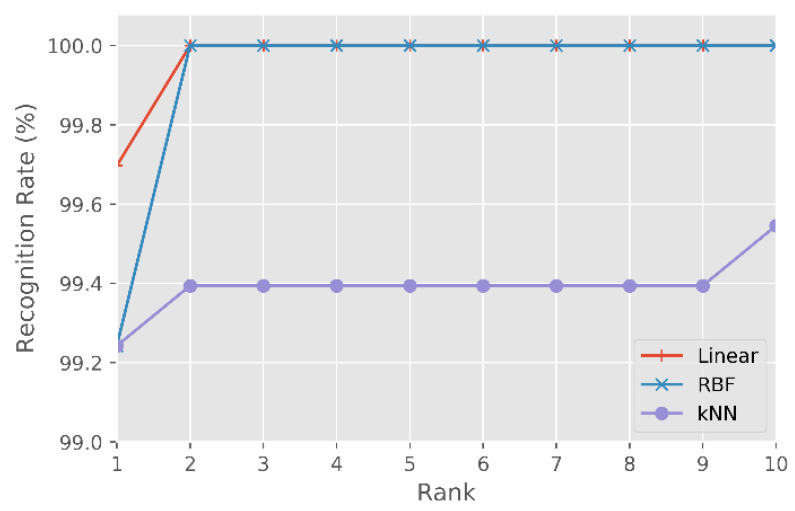
CMC curve for the SVM-Linear, SVM-RBF and kNN classifiers, for the Tufts multispectral database test set.

Figure 7 shows the CMC curve, for the three classifiers, for the first 10 classifications (e.g., rank-10) for the Tufts database [13].

Performing a comparative analysis between the three classifiers through the rank-1 values, the SVM-Linear classifier is the one that obtains the best results, with a rank-1 score of 99.7%. Respectively, the SVM-RBF and kNN classifiers scored 99.2% for the same set of images.

Figure 7 also shows that for rank-2 and regardless of the kernel used in the SVM classifier, an identification rate of 100% is achieved, i.e., all the facial images in the test set were correctly identified. Instead, the kNN classifier only achieves an identification rate of 100% in rank-102.

Based on the previous results, we consider the SVM-Linear classifier as the most suited to these databases. Thus, our proposed methodology for multispectral facial recognition is composed of the pre-trained LightCNN as base DCNN, fine-tuning the layers ({1–3} + FCL), to produce a set of 256-d embeddings classified by the SVM-Linear classifier.

Table 4 compares the results of the proposed methodology with other methodologies described in the literature. In bold the method that produced the best score in rank-1 is highlighted.

Table 4 shows that the proposed methodology produces much better results compared to the other methodologies. It should be noted that the Tufts multispectral database used for DCNN training was cleaned by us. Other authors do not specify whether, or not, they have cleaned the database. We note that some of the images on this database were excluded due to very bad quality. Note that 53 facial images were excluded from the Tufts multispectral database. However, 27 of these facial images were excluded because two persons did not have images in all spectral bands, namely VIS, NIR and LWIR. However, even if all the 26 excluded images were wrongly classified by our method, a rank-1 score of 95.9% would be obtained, which is still higher than the state-of-the-art for this database. 

It should be noted that, as the Tufts database was made available to the public only in 2020, the number of researchers using this database is still small.

###### Results Using the CASIA NIR-VIS 2.0 Database

After processing the CASIA NIR-VIS 2.0 database test set with the classifiers (i) SVM-Linear, (ii) SVM-RBF and (iii) kNN, the following values were obtained in rank-1: (i) 99.8%, (ii) 99.8% and (iii) 99.7%. 

**Figure 8:**
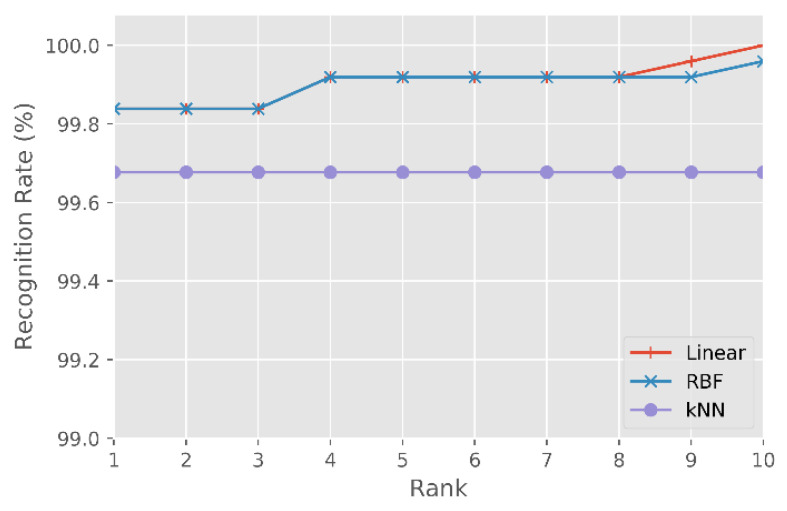
CMC curve for the SVM-Linear, SVM-RBF and kNN classifiers, for the CASIA NIR-VIS 2.0 test set.

Figure 8 shows the CMC curve for the three classifiers in the first 10 classifications. Both SVM classifiers, regardless of the kernel used, obtain a score of 99.8%. In comparison, the kNN classifier achieves a rank-1 score of 99.7%.

Figure 8 shows that after rank-8 the SVM-Linear classifier achieves a higher identification rate than the SVM-RBF. SVM-Linear and SVM-RBF classifiers get an identification rate of 100% for rank-10 and rank-12, respectively. 

Table 4 shows the results obtained using the proposed methodology and using other methodologies described in the literature. The method that obtained the best score in rank-1 is highlighted in bold. Note that the table is listed by year of publication and not by the rank-1 values.

The proposed methodology uses the LightCNN as base DCNN, adapting the layers ({1–3} + FCL), to produce a set of 256-d embeddings, which are later classified by the SVM-Linear classifier.

From the results shown in Table 5 the proposed methodology obtains superior results in rank-1 when compared to the other methods described in the state-of-the-art. 

The most recent work that uses the CASIA NIR-VIS 2.0 database is Bae et al. [28] that obtained a rank-1 score of 99.4%, lower than the result obtained by our proposed methodology (99.8%).

After a detailed analysis of Table 5, it is possible to see that LightCNN [11] base methodology obtained a rank-1 score of 96.7%. Through the proposed methodology, it was possible to improve the rank-1 score by 3.1%.

## 5. Conclusions

In this work, a multispectral facial recognition system was proposed. This system takes advantage of multispectral images to obtain better facial recognition results. The system is composed of four modules: image processing, presentation attack detector, facial processing, and facial recognition. 

The main contribution of the paper is a facial recognition architecture based on fine-tuning, with domain specific data, the initial layers of DCNNs pre-trained in large face datasets of images in the visible spectrum. These DCNNs extract feature embeddings for each channel of the multispectral image. A final fully connected layer fuses the information of the several channels in a 256-dimensional embedding, that is then classified by a linear SVM. Instead of traditional transfer learning methods, that fine tune the last layers of the networks to match the required task, we fine tune the initial layers to match the characteristics of the input spectral bands. 

Extensive studies in the multispectral databases demonstrated the superiority of the proposed methodology. The obtained results show better rank-1 scores than the state-of-the-art methods in the multispectral databases Tufts and CASIA NIR-VIS 2.0, respectively, 99.7% and 99.8%. Previous works present best scores in rank-1 for these databases of 94.5% and 99.4%, respectively.

Additionally, we explored the characteristics of multiple infrared spectral channels to develop a presentation attack detector. Our module uses a skin detector empirically tuned in multispectral images to create a binary map. With this map, a comparison is made with the facial landmarks to obtain the percentage of facial landmarks that are skin. Skin detectors based on YCbCr and HSV color spaces are used to compare with our multispectral skin detector. The multispectral presentation attack detector achieves better results (83%) than those that use only visible images (13%).

## Figures and Tables

**Table 1:** Parameters used in the training procedure for each multispectral database.

Parameters	Database	
	Tufts	CASIA NIR-VIS 2.0
Batch size	16	32
Optimization algorithm	Adam	Adam
Learning rate	0.001	0.001
Epoch number	10	50

**Table 2:** Optimal values for each hyperparameter and their mean rank-1 and standard deviation using the Tufts database.

Classifier	Regularization Parameter (*C*)	Kernel Coefficient (*Υ*)	Number of Neighbors (*k*)	Rank-1 (Mean Value)	Rank-1 (Standard Deviation)
({1–3} + FCL) + SVM-Linear	10^−2^	-	-	99.89%	0.09%
({1–3} + FCL) + SVM-RBF	10^+1^	10^−4^	-	99.89%	0.09%
({1–3} + FCL) + kNN	-	-	1	99.54%	0.35%

**Table 3:** Optimal values for each hyperparameter and its mean rank-1 and standard deviation using the CASIA NIR-VIS 2.0 database.

Classifier	Regularization Parameter (*C*)	Kernel Coefficient (*Υ*)	Number of Neighbors (*k*)	Rank-1 (Mean Value)	Rank-1 (Standard Deviation)
({1–3} + FCL) + SVM-Linear	10^−3^	-	-	99.86%	0.06%
({1–3} + FCL) + SVM-RBF	10^+1^	10^−5^	-	99.86%	0.06%
({1–3} + FCL) + kNN	-	-	1	99.63%	0.25%

**Table 4:** Results produced through the proposed methodology when compared with the state-of-the-art for the tufts database.

Method	Rank-1	Year of Publication
TR-GAN [18]	88.7%	2019
Circular HOG [19]	94.5%	2020
Proposed methodology	**99.7%**	2020

**Table 5:** Results obtained through the proposed methodology when compared with the state-of-the-art for the CASIA NIR-VIS 2.0 database.

Method	Rank-1	Year of Publication
CDFL [20]	71.5%	2015
MCA [21]	69.1%	2016
MTC-ELM [22]	89.1%	2017
CEFDA [23]	85.6%	2017
Oh et al. [24]	97.5%	2017
LightCNN [11]	96.7%	2018
MDNDC [4]	98.9%	2019
Peng et al. [25]	96.7%	2019
DSU [10]	96.3%	2019
WCNN [26]	98.7%	2019
DDFLJM [27]	98.8%	2019
Peng et al. [28]	98.7%	2019
CFC [29]	98.6%	2019
CycleGAN [30]	99.4%	2020
Proposed methodology	99.8%	2020

## Data Availability

For access to the CASIA NIR-VIS 2.0 database [8] a release agreement was filled and sent to cbsr-request@authenmetric.com (accessed on 30 June 2021), then access to the database was presented. For access to the Tufts Face database a release agreement was filled in http://tdface.ece.tufts.edu/ (accessed on 30 June 2021), then access to the database was presented.

## References

[1] Jain A.K., Ross A.A., Nandakumar K. (2011). Introduction to Biometrics.

[2] Munir R., Khan R.A. (2019). An extensive review on spectral imaging in biometric systems: Challenges & advancements. J. Vis. Commun. Image Represent..

[3] Zhang W., Zhao X., Morvan J., Chen L. (2019). Improving Shadow Suppression for Illumination Robust Face Recognition. IEEE Trans. Pattern Anal. Mach. Intell..

[4] Hu W.P., Hu H.F., Lu X.L. (2019). Heterogeneous Face Recognition Based on Multiple Deep Networks with Scatter Loss and Diversity Combination. IEEE Access.

[5] Bento N.A., Silva J.S., Bioucas-Dias J. (2016). Detection of Camouflaged People. Int. J. Sens. Netw. Data Commun..

[6] Silva J.S., Guerra I.F.L., Bioucas-Dias J., Gasche T. (2019). Landmine Detection Using Multispectral Images. IEEE Sens. J..

[7] Chambino L.L., Silva J.S., Bernardino A. (2020). Multispectral Facial Recognition: A Review. IEEE Access.

[8] Li S., Yi D., Lei Z., Liao S. The CASIA NIR-VIS 2.0 Face Database. Proceedings of the IEEE Conference on Computer Vision and Pattern Recognition Workshops.

[9] George A., Mostaani Z., Geissenbuhler D., Nikisins O., Anjos A., Marcel S. (2020). Biometric Face Presentation Attack Detection With Multi-Channel Convolutional Neural Network. IEEE Trans. Inf. Forensics Secur..

[10] Pereira T.D., Anjos A., Marcel S. (2019). Heterogeneous Face Recognition Using Domain Specific Units. IEEE Trans. Inf. Forensics Secur..

[11] Wu X., He R., Sun Z., Tan T. (2018). A Light CNN for Deep Face Representation with Noisy Labels. IEEE Trans. Inf. Forensics Secur..

[12] Fu X., Lu J., Zhang X., Yang X., Unwala I. Intelligent In-Vehicle Safety and Security Monitoring System with Face Recognition. Proceedings of the IEEE International Conference on Computational Science and IEEE International Conference on Embedded and Ubiquitous Computing Engineering.

[13] Panetta K., Wan Q., Agaian S., Rajeev S., Kamath S., Rajendran R., Rao S.P., Kaszowska A., Taylor H.A., Samani A. (2020). A Comprehensive Database for Benchmarking Imaging Systems. IEEE Trans. Pattern Anal. Mach. Intell..

[14] Mishkin D., Sergievskiy N., Matas J. (2017). Systematic Evaluation of Convolution Neural Network Advances on the Imagenet. Comput. Vis. Image Underst..

[15] Goodfellow I., Bengio Y., Courville A. (2016). Deep Learning.

[16] Forman G., Scholz M. (2010). Apples-to-Apples in Cross-Validation Studies: Pitfalls in Classifier Performance Measurement. ACM Sigkdd Explor. Newsl..

[17] Tsamardinos I., Rakhshani A., Lagani V. (2015). Performance-Estimation Properties of Cross-Validation-Based Protocols with Simultaneous Hyper-Parameter Optimization. Int. J. Artif. Intell. Tools.

[18] Kezebou L., Oludare V., Panetta K., Agaian S. TR-GAN: Thermal to RGB face synthesis with generative adversarial network for cross-modal face recognition. Proceedings of the SPIE Mobile Multimedia/Image Processing, Security, and Applications.

[19] Rajeev S., Shreyas K., Wan Q., Panetta K., Agaian S. (2019). Illumination Invariant NIR Face Recognition Using Directional Visibility. Electron. Imagingimage Process. Algorithms Syst..

[20] Jin Y., Lu J.W., Ruan Q.Q. (2015). Coupled Discriminative Feature Learning for Heterogeneous Face Recognition. IEEE Trans. Inf. Forensics Secur..

[21] Li Z.F., Gong D.H., Li Q., Tao D.C., Li X.L. (2016). Mutual Component Analysis for Heterogeneous Face Recognition. ACM Trans. Intell. Syst. Technol..

[22] Jin Y., Li J., Lang C.Y., Ruan Q.Q. (2017). Multi-task clustering ELM for VIS-NIR cross-modal feature learning. Multidimens. Syst. Signal Process..

[23] Gong D.H., Li Z.F., Huang W.L., Li X.L., Tao D.C. (2017). Heterogeneous Face Recognition: A Common Encoding Feature Discriminant Approach. IEEE Trans. Image Process..

[24] Oh B.S., Oh K., Teoh A.B.J., Lin Z.P., Toh K.A. (2017). A Gabor-based network for heterogeneous face recognition. Neurocomputing.

[25] Peng C.L., Wang N.N., Li J., Gao X.B. (2019). DLFace: Deep local descriptor for cross-modality face recognition. Pattern Recognit..

[26] He R., Wu X., Sun Z.N., Tan T.N. (2019). Wasserstein CNN: Learning Invariant Features for NIR-VIS Face Recognition. IEEE Trans. Pattern Anal. Mach. Intell..

[27] Hu W.P., Hu H.F. (2019). Discriminant Deep Feature Learning based on joint supervision Loss and Multi-layer Feature Fusion for heterogeneous face recognition. Comput. Vis. Image Underst..

[28] Peng C.L., Wang N.N., Li J., Gao X.B. (2019). Re-Ranking High-Dimensional Deep Local Representation for NIR-VIS Face Recognition. IEEE Trans. Image Process..

[29] He R., Cao J., Song L., Sun Z., Tan T. (2019). Adversarial cross-spectral face completion for NIR-VIS face recognition. IEEE Trans. Pattern Anal. Mach. Intell..

[30] Bae H.B., Jeon T., Lee Y., Jang S., Lee S. (2020). Non-Visual to Visual Translation for Cross-Domain Face Recognition. IEEE Access.

